# New Evidence of Rubber-Derived Quinones in Water, Air, and Soil

**DOI:** 10.1021/acs.est.1c07376

**Published:** 2022-03-22

**Authors:** Guodong Cao, Wei Wang, Jing Zhang, Pengfei Wu, Xingchen Zhao, Zhu Yang, Di Hu, Zongwei Cai

**Affiliations:** State Key Laboratory of Environmental and Biological Analysis, Department of Chemistry, Hong Kong Baptist University, Kowloon, Hong Kong SAR 999077, China

**Keywords:** *p*-phenylenediamines, rubber-derived quinones, 6PPD-quinone, human health

## Abstract

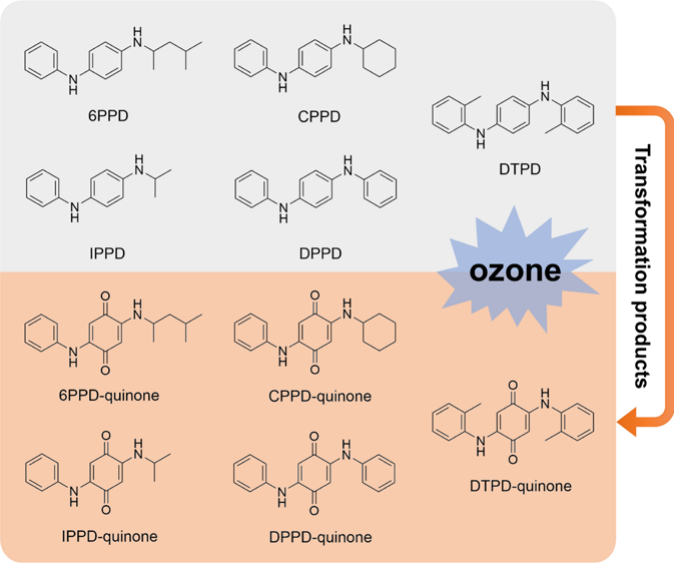

*p*-Phenylenediamines (PPDs) have been extensively used in the rubber industry and found to be pervasive in various environmental compartments for decades, while their transformation products and associated ecological and human health risks remain largely unknown. Herein, we developed and implemented a mass spectrometry-based platform combined with self-synthesized standards for the investigation of rubber-derived quinones formed from PPD antioxidants. Our results demonstrated that five quinones are ubiquitously present in urban runoff, roadside soils, and air particles. All of the identified sources are closely related to mankind’s activities. Among the identified quinones, *N*-(1,3-dimethylbutyl)-*N*′-phenyl-*p*-phenylenediamine quinone has been recently found to be highly toxic, causing acute mortality of coho salmon in the Pacific Northwest. Ultrahigh-performance liquid chromatography coupled with triple quadrupole mass spectrometry was then applied for quantification of the five quinones and their corresponding PPD antioxidants. The results revealed interesting distinct distribution and concentration patterns of PPD-derived quinones in different environmental matrices. Daily intake rates of these quinones in a compact city of Hong Kong were estimated to be varied from 1.08 ng/(kg·day) for adults to 7.30 ng/(kg·day) for children, which were higher than the exposure levels of their parent compounds. Considering the prevalence of the use of rubber products, the outcome of this study strongly suggests for additional toxicological studies to investigate potential ecological and human health risks of the newly discovered quinones.

## Abstract 2

Five *p*-phenylenediamine-derived quinones have been identified to be pervasive in roadside soil, runoff water, and air particles, while their health risks remain elusive. Research findings demonstrate the coexistence of five quinones from globally ubiquitous *p*-phenylenediamine antioxidants in urban runoff, roadside soils, and air particles.

## 1 Introduction

Our century has witnessed an unprecedented growth in the production of man-made chemicals that have been extensively used in foods, medicines, and industrial materials with the goal of improving the standard of living.^1−3^ However, a substantial proportion of the chemicals may be released into different environmental compartments, undergo complex degradation and transformation processes, and generate new toxicants.^4−7^

*N*,*N*′-Substituted *p*-phenylenediamines (PPDs) are manufactured and widely used as antioxidants and antiozonants in the industry for production of tires, belts, hoses, and cables.^8−10^ The chemicals provide superior capacity for protection of rubber and its products against heat degradation, breaking down, and ozone cracking.^8,11,12^ However, the vast number of rubber products have resulted in an incredible release of PPDs and related degradation products into the environment. Various PPDs, as indicated in Table 1, such as *N*-(1,3-dimethylbutyl)-*N*′-phenyl-*p*-phenylenediamine (6PPD), *N*,*N*′-bis(1,4-dimethylpentyl)-*p*-phenylenediamine, *N*-phenyl-*N*′-cyclohexyl-*p*-phenylenediamine (CPPD), *N*-isopropyl-*N*′-phenyl-1,4-phenylenediamine (IPPD), *N*,*N*′-di(*o*-tolyl)-*p*-phenylenediamine (DTPD), and *N*,*N*′-diphenyl-*p*-phenylenediamine (DPPD), have been detected in different environmental matrices, including airborne particles, water, and sediments,^13−21^ which has yielded grave concerns regarding their potential threats to the environment and human health. A study by Prosser *et al.* indicated that 6PPD could induce species-specific toxicity in aquatic organisms.^14^ Matsumoto *et al.* demonstrated that exposure of DPPD enabled prolonged gestation and caused dystocia in female rats.^22^ In addition, IPPD and CPPD detected in rubber shoes and wrist straps were found to be associated with the development of allergic contact dermatitis in farmers and plumbers.^23−25^

**Table 1:**
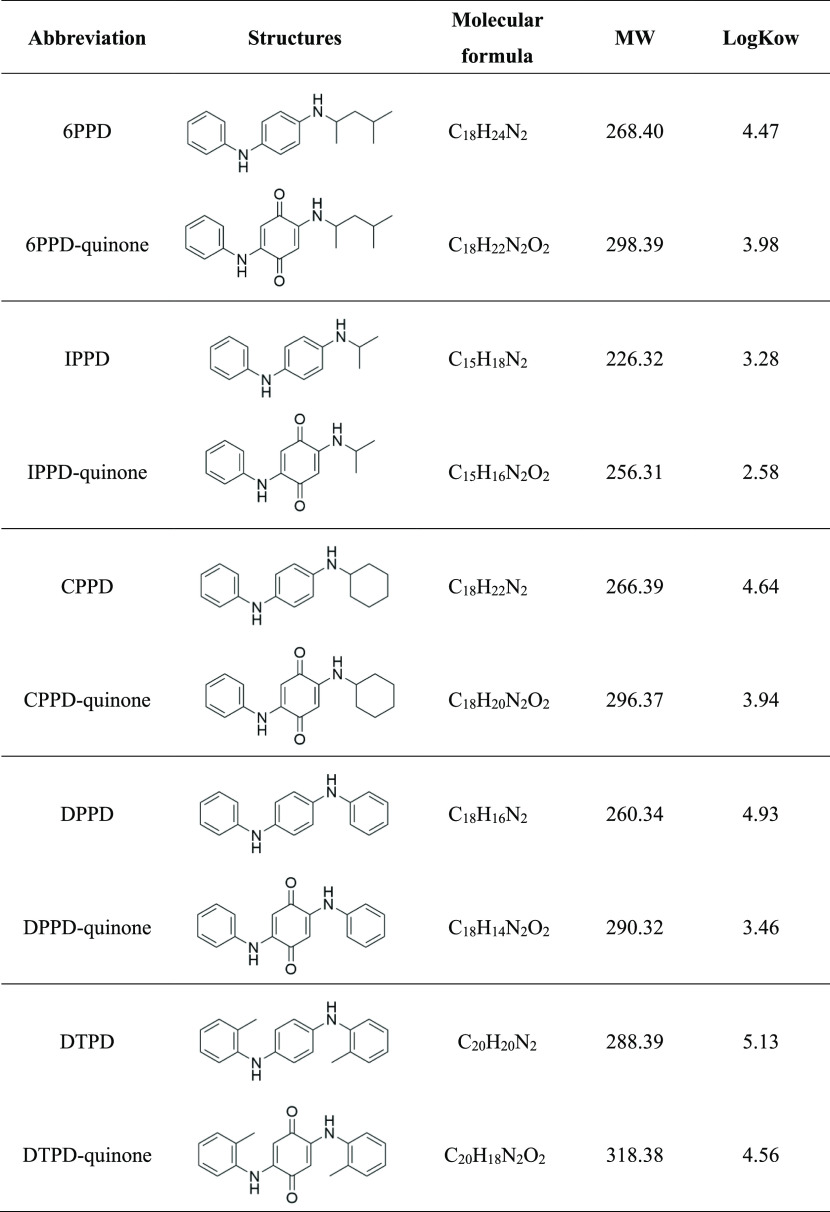
Abbreviations, Structures, Molecular Weights (MW), and Physicochemical Properties of PPDs and Their Derived Quinone Analogues Discussed in This Study

In a recent study, Tian and colleagues identified *N*-(1,3-dimethylbutyl)-*N*′-phenyl-*p*-phenylenediamine quinone (6PPD-quinone) as a transformation product of 6PPD.^26^ The new toxicant was found to be pervasive in roadway runoff and runoff-affected receiving waters, causing acute mortality of coho salmon in the Pacific Northwest before they spawn in freshwater streams, a phenomenon called the “urban runoff mortality syndrome”. The seminal paper and results have aroused significant interest and concern regarding the occurrence and concentration of 6PPD-quinone in other environmental matrices. Huang *et al.* demonstrated the presence of 6PPD-quinone in dust samples by using mass spectrometry (MS),^27^ despite the 6PPD-quinone standard not being the available standard used for quantitation at that time. Later on, Johannessen *et al.* measured the amount of 6PPD-quinone in urban receiving waters collected from tributaries of Lake Ontario.^28^ In our recent study, we reported the ubiquitous distributions of 6PPD-quinone and its parent compound in fine particulate matters (PM2.5).^29^ These results, along with the findings by Tian *et al.*, provided compelling evidence indicating the presence of 6PPD-quinone in the environment.

In addition to 6PPD, we suspect that other PPDs may undergo similar transformation reactions and generate the corresponding quinones. To test this hypothesis, we developed and implemented an MS-based platform for the investigation of the presence and the levels of rubber-derived quinones in water, air, and soil samples. Versatile mass spectrometric techniques, including data-dependent acquisition, selected ion monitoring, and scheduled selected reaction monitoring (SRM) modes, in combination with synthesis of the targeted quinone compounds, have been adopted for uncovering the environmental pollutants. Our results demonstrated that five rubber-derived quinones, including 6PPD-quinone, existed in the environment as the transformation products of PPD antioxidants. Such findings greatly expand the existing knowledge of PPD-derived quinones and thus may provide opening clues and promote further investigations on their environmental fate and ecological risks in the near future.

## 2 Material and Methods

### 2.1 Chemicals and Reagents

PPD standards and 6PPD-quinone were purchased from J&K Scientific Ltd. (Hong Kong, China) and TCI Chemicals (Tokyo, Japan). The surrogate standard of diphenylamine-*d*_10_ was purchased from Toronto Research Chemicals (Burlington, Canada). Chemicals used for synthesis of PPD-derived quinones and the internal standard of 6PPD-quinone-*d*_5_, including aniline, *o*-toluidine, isopropyl amine, cyclohexylamine, 1,3-dimethylbutylamine hydrochloride, 1,4-benzoquinone, and aniline-*d*_5_, were obtained from Sigma-Aldrich (Hong Kong, China) and Toronto Research Chemicals (Burlington, Canada). The purities of the synthesized standards were estimated to be ∼95–98% on the basis of the total ^1^H nuclear magnetic resonance (NMR) integral. High-performance liquid chromatography (HPLC)-grade acetonitrile, methanol, and dichloromethane were purchased from VWR Chemicals (Fontenay-sous-Bois, France).

### 2.2 Sampling and Sample Pretreatment

The soil, water, and atmospheric particle samples were collected separately and processed independently for all steps. Roadside soil samples were collected in New Territories and Kowloon, Hong Kong during August to September 2021. Urban runoff water samples were collected in a dense traffic urban area in Kowloon, Hong Kong in August 2021. 24 h atmospheric particles were collected on quartz fiber filters using a moderate-volume air sampler during September 2020 to August 2021 in the campus of Hong Kong Baptist University. More details about sample collection, including sampling locations, rain events, and holding time, are given in Table S1.

For soils, 100 mg of the sample (dry weight) was spiked with 50 ng of the surrogate standard (diphenylamine-*d*_10_) and ultrasonicated twice for 15 min in 3 mL of dichloromethane. Another ultrasonic extraction was performed in 3 mL of acetonitrile for 15 min. The extracts were combined and concentrated to dryness via nitrogen purge. The dried extract was redissolved in 100 μL of acetonitrile, spiked with 50 ng of the internal standard (6PPD-quinone-*d*_5_), and filtered through a 0.45 μm navigator nylon organic filter membrane before the instrumental analysis. The pretreatment of air particle samples was consistent with that of the roadside soils with mirror modification. Half of the filters were cut and placed into a 15 mL glass tube, spiked with 50 ng of the surrogate standard, and then ultrasonicated twice for 15 min with 5 mL of dichloromethane. After repeating the ultrasonic extraction for another 15 min with 5 mL of acetonitrile, the extracts were combined and concentrated to near dryness via nitrogen purge. The dried extracts were redissolved in 100 μL of acetonitrile with 50 ng of the internal standard and filtered prior to the MS detection. For water samples, 50 mL of the runoff water was filtered by a glass microfiber filter (1.2 μm) to remove the possible impurities, then spiked with 50 ng of the surrogate standard, and acidified with 2% formic acid by volume. The extraction was conducted using a hydrophilic–lipophilic balance solid-phase extraction (SPE) cartridge (60 mg, 3 mL, Waters) at a flow rate of 5–8 mL/min. The SPE cartridges were preconditioned with 1 mL of methanol and 1 mL of deionized water, respectively. After the sample was loaded, the cartridge was drenched with 1 mL of water with 5% methanol (v/v), vacuum-dried for 15 min, and eluted with 3 mL of a methanol–dichloromethane (1:9, v/v) mixed solvent. The elute was blown to dryness, redissolved in 100 μL of acetonitrile containing 50 ng of the internal standard, and then filtered through a 0.45 μm navigator nylon organic filter membrane for instrumental analysis.

### 2.3 Instrumental Analysis

#### 2.3.1 Ultrahigh-performance LC Orbitrap Mass Spectrometry

An UltiMate 3000 ultrahigh-performance LC (UHPLC) system coupled with a Q Exactive mass spectrometer (Thermo Fisher Scientific, USA) was used for chemical screening. The mass analyzer was operated alternately in the full scan mode (*m*/*z* 80–600, mass resolution, 35,000) and data-dependent acquisition (mass resolution, 17,500) mode. Electrospray ionization (ESI) conditions, including capillary voltage, capillary temperature, probe heater temperature, and sheath gas flow, were optimized to be 3.6 kV in the positive ion mode, 350 °C, 320 °C, and 40 arbitrary units, respectively. Chromatographic separation was performed on an Acquity HSS T3 column (1.8 μm, 2.1 × 100 mm) with mobile phases consisting of (A) 0.1% formic acid in water (by volume) and (B) acetonitrile. The mobile phase flow rate was set as 0.3 mL/min. The elution started with 2% B (0–1 min), increased to 100% B (1–19 min), held for 3 min (19–22 min), then back to 2% B, and rebalanced for 3 min. The selected ion monitoring mode was used for targeted examination of PPDs and quinones, in which the mass resolution and mass isolation window were set as 35,000 and ±0.8 *m*/*z*, respectively.

#### 2.3.2 UHPLC Triple Quadrupole Mass Spectrometry

An UltiMate 3000 UHPLC system interfaced with a triple-quadrupole mass spectrometer (Thermo Fisher Scientific, USA) was used for target quantitation of PPDs and quinones. The mass analyzer was operated at a scheduled SRM mode, in which the precursor ion isolation window and detection window were set as 0.7 *m*/*z* and 60 s, respectively. The parameters in the ESI source and chromatography conditions were kept the same as those descried above. Details of SRM transitions, including quantifier ions, qualifier ions, collision energy values, and calibration curves, are summarized in Table S2.

### 2.4 Quality Control and Quality Assurance

To determine the method recovery, 50 ng of mixed standards including PPDs and *p*-phenylenediamine-derived quinones (PPD-Qs) were spiked into 100 mg of diatomaceous earth, half of the blank filter (pre-baked), 50 mL of deionized water, and 50 mL of runoff water, respectively, to process the entire extraction procedure in triplicate. The results were summarized in Table S2. The reproducibility of the method was assessed by performing three runs of each type of the sample, and the relative standard deviations for the targeted chemicals were in the range of 3.4 to 7.7%. The instrument detection limit (IDL) and instrument quantification limit (IQL) were defined as 3 and 10 times the standard deviation of the signal-to-noise ratio (S/N), respectively. To evaluate possible contamination caused by sampling and pretreatment procedures, field blank samples including deionized water, diatomaceous earth, and quartz fiber filters were transported, stored, and extracted in the same manner as that of runoff water, soil, and atmospheric particle samples, respectively, and these controls were determined as zero. The blank spike recoveries of PPDs and their quinones were in the range of 75 to 93% in water, 70 to 113% in soil, and 74 to 96% in atmospheric particles, respectively. The measured concentrations of real samples were not normalized to the observed recoveries.

### 2.5 Data Analysis and Exposure Assessment

The MS data were processed by using Xcalibur software (Thermo Scientific), which encompassed feature extraction, peak integration, and background subtraction for identification and target quantitation.^30^ Statistical analyses for calculation of the geometric mean, concentration range, and average composition were conducted using the utility of SPSS 11.0 (IBM, SPSS Inc.). The physicochemical properties of the natural form of PPDs and quinones were calculated using EPI Suite software (V.4.11, US EPA), and the results are given in Table S3. The estimated daily intake of PPDs and their corresponding quinones was calculated following the equations below
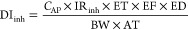
1
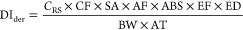
2
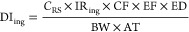
3where DI_inh_, DI_der_, and DI_ing_ represent the daily intake doses from inhalation through ambient air, dermal absorption with soil, and oral ingestion *via* soil dust particles, respectively. *C*_AP_ and *C*_RS_ are the total concentrations of PPDs (∑PPDs) and PPD-Qs (∑PPD-Qs) in air particles (ng/m^3^) and roadside soils (ng/kg), respectively; IR_inh_ and IR_ing_ are the inhalation rates (m^3^/day) for air particles and ingestion rate (mg/day) for roadside soils, respectively; EF is the exposure frequency (days/year); ED is the exposure duration (years); BW is the body weight (kg); AT is the average time during exposure (days); CF is the conversion factor (10^–6^ kg/mg); SA is the skin surface area available for contact (cm^2^); AF is the soil-to-skin adherence factor (mg/cm^2^); and ABS is the absorption factor (unitless). The parameters used to evaluate the DI values for children and adults were in accordance with the risk assessment guidance^31−33^ and relative studies,^34,35^ as shown in Table S4.

## 3 Results and Discussion

### 3.1 Identification of Rubber-Derived Quinones in Environmental Samples

**Figure 1:**
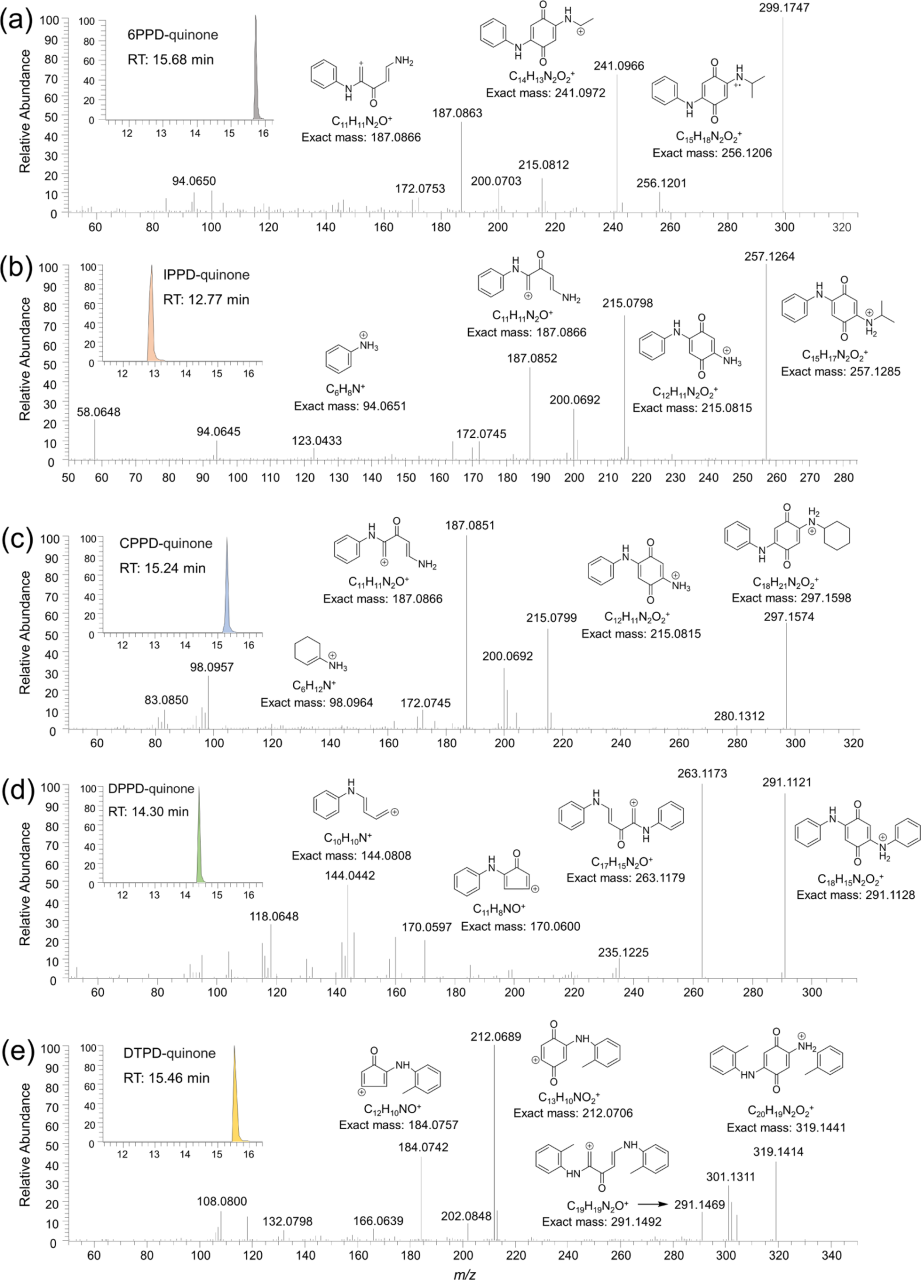
Extracted ion chromatograms and MS/MS spectra of [M + H]^+^ ions of 6PPD-quinone (a), IPPD-quinone (b), CPPD-quinone (c), DPPD-quinone (d), and DTPD-quinone (e) using UHPLC Orbitrap MS analysis.

The high-resolution MS-based “global” profiling method was first applied for screening chemical profiles of the collected environmental samples. The method operated at the full scan and data-dependent acquisition modes enabled obtaining full scan MS and tandem MS spectra of molecules for identification of the unknown.^36,37^ On the basis of the previous studies, we first examined the occurrence of 6PPD-quinone in roadside soils. As indicated in Figure S1, searching for the pseudo-molecular ion of [C_18_H_25_N_2_]^+^ in the extracts yielded a single chromatographic peak at *m*/*z* 299.1747, which differed from the theoretical protonated molecular ion of 6PPD-quinone by 2.34 ppm. In parallel, its MS^2^ spectrum gave fragment ions at *m*/*z* 256.1201 and 241.0966, which is rationalized by the sequential losses of C_3_H_7_ and C_4_H_10_ from the parent ion, respectively (Figure 1a). In addition, a pair of characteristic fragment ions at *m*/*z* 215.0812 and 187.0863, suffering from a neutral loss of 28 Da, and a base peak with a low intensity at *m*/*z* 94.0650 indicated the existence of carbonyl and aniline groups in the molecular structure, which agreed well with the tandem mass spectrum of 6PPD-quinone determined by Tian *et al.*(26) The same chromatographic peaks were also observed in the detection of runoff water and air particles (Figure S1), consistent with that of the commercial standard of 6PPD-quinone. These findings so far demonstrated that 6PPD-quinone is ubiquitously present in various environmental media, including runoff water, roadside soil, and air particles.

Next, we interrogated whether other types of rubber-derived quinones may also be detectable in these environmental samples. Herein, we applied a targeted selected ion monitoring method for sensitive and specific measurement of PPD antioxidants and their possible quinone products *via* the proposed formation pathway in Figure S2. Through a review of published reports and literature precedents,^13,15,16,38,39^ a total 16 PPD antioxidants and their presumptive quinone compounds were selected *a priori* for suspect screening. It is intriguing to note that five chromatographic peaks implying the presence of PPD-Qs existed in the extracts of samples. The first one was observed at *m*/*z* 257.1264 with a retention time of 12.8 min (Figure 1b), where its MS^2^ spectrum exhibited characteristic fragment ions at 215.0798, 187.0852, and 94.0645. These fragment ions along with their neutral losses were in line with the fragmentation mechanism of 6PPD-quinone, as previously described, indicating that this compound belongs to the homologous series of 6PPD-quinone. The same fragmentation pattern was also observed in another chromatographic peak at *m*/*z* 297.1574, as expected for CPPD-quinone (Figure 1c), which has a cyclohexylamine substituent on one side of the quinone ring. It worth noting that the fragment ions originating from DPPD-quinone were not in agreement with the fragmentation patterns determined by IPPD-quinone and CPPD-quinone (Figure 1d). The first fragment ion was observed at *m*/*z* 263.1173 with a neutral loss of 28 Da, and a second fragment ion was observed at *m*/*z* 235.1225 with a neutral loss of 56 Da, indicating consecutive losses of two carbonyl moieties from the parent ion. This difference can be rationalized due to the symmetric phenyl substituents of DPPD-quinone. In analogy to this observation, a chromatographic peak at *m*/*z* 319.1414 with a retention time of 15.5 min, which corresponds to the anticipated quinone of DTPD, was also detected in the sample extracts. Its MS^2^ spectrum exhibited a base peak at *m*/*z* 291.1469, revealing the loss of the carbonyl group, followed by the formation of a characteristic fragment ion at *m*/*z* 212.0689 upon the loss of the toluidine moiety (Figure 1e). To further confirm the identity of these tentatively compounds, we designed a synthetic route for the synthesis of PPD-derived quinones due to the lack of commercial standards (Figure S3). Among the compounds, IPPD-quinone, CPPD-quinone, and 6PPD-quinone were synthetized *via* a stepwise addition reaction, whereas DPPD and DTPD were prepared *via* a one-step oxidative addition of *para*-quinone with aniline and *o*-toluidine, respectively (Supporting Information). Of note, this approach enabled synthesis of isotope-labeled 6PPD-quinone-*d*_5_ by replacing aniline with aniline-*d*_5_, which was used as an internal standard for accurate quantification of PPD-Qs in different environmental matrices. The synthetic compounds were well-characterized using NMR spectroscopy, infrared spectroscopy, and ESI-MS (Supporting Information). These synthetic compounds were found to have high purity, as exemplified by the ^1^H NMR spectra of 6PPD-quinone and 6PPD-quinone-*d*_5_ (Figure S4), among which the synthesized 6PPD-quinone exhibited an identical retention time and MS^2^ spectrum and approximately equal peak-area responses to its commercial standard (Figure S5). On the basis of the total ^1^H NMR integral, we estimated the purity of these synthesized standards to be ∼95–98%. The identities of the tentatively identified quinones were confirmed by matching the monoisotopic precursor, retention time, and MS^2^ spectra with those of the synthetic standards, which provided compelling evidence indicating the ubiquitous presence of the five PPD-derived quinones in the environment.

### 3.2 Occurrence and Allocation of Rubber-Derived Quinones in Multiple Environmental Media

**Figure 2:**
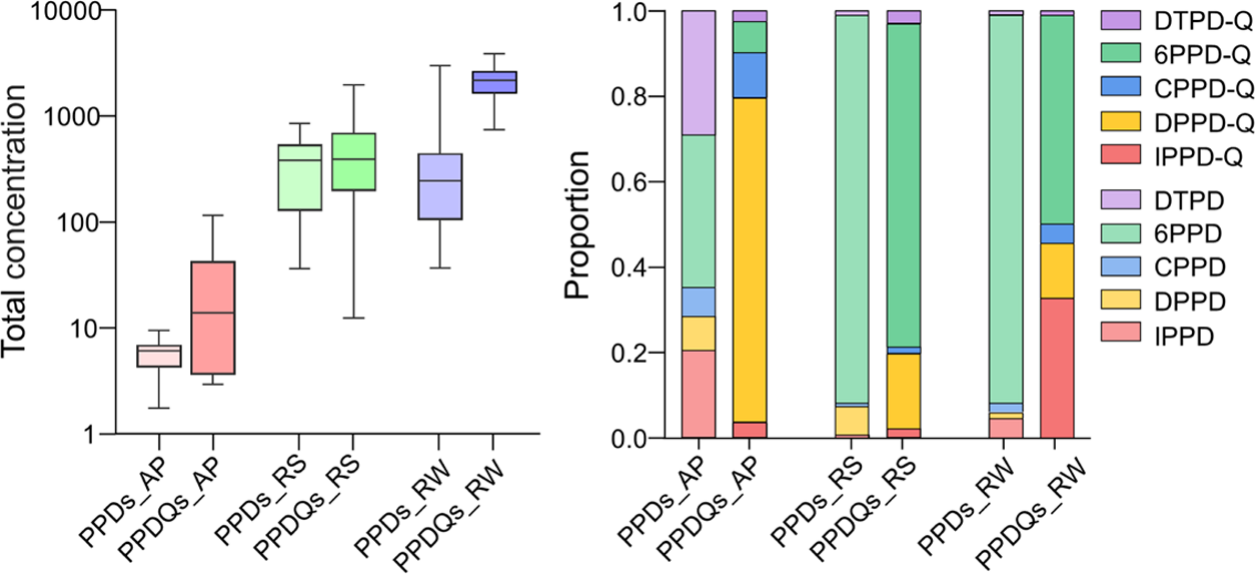
Total concentration and composition profiles of PPDs and PPD-Qs in multiple environmental media. Concentrations are given in picogram per cubic meter for air particles (AP), nanogram per gram for roadside soils (RS), and microgram per liter for runoff water (RW).

For a better understanding of the environmental relevance of rubber-derived quinones and evaluating the impact of their possible emissions to humans, we established an UHPLC triple quadrupole MS method for target quantitation of the five PPD-Qs and their parent compounds. The precursor–product ion pairs were extracted from the standard MS^2^ spectra (Figure 1), where the most intense fragment ions were selected and optimized as the quantifier and qualifier ions, respectively. Figure S6 shows the SRM chromatograms of five PPD-Qs detected in runoff water, soil, and air particles in comparison to those of the synthesized standards. As indicated in Table 2, all these PPD antioxidants and PPD-Qs were detectable in various environmental media. DPPD-quinone and 6PPD-quinone exhibited a high detection frequency (100%) in runoff water, air particles, and roadside soils, indicating their widespread occurrence in the environment. The concentrations of total PPD-Qs in air particles varied in the range of 2.52–196 pg/m^3^ (a median of 4.92 pg/m^3^), comparable to the levels of their patent compounds (1.75–9.41 ng/g) (a median of 6.07 pg/m^3^). In comparison with other organic contaminants, including organophosphate flame retardants^40^ and amino antioxidants,^41^ the pollution levels of PPD-Qs and PPDs were not high. By contrast, the concentrations of total PPD-Qs (0.74–3.87 μg/L, a median of 2.17 μg/L) determined in runoff water were higher than the appearance of their patent compounds (0.04–3.00 μg/L, a median of 0.24 μg/L). This phenomenon can be partly explained by the differences in physicochemical properties and fate of PPD-Qs *via* complex transformation processes. Among these detected quinones, we noticed that DPPD-quinone is particularly abundant in the air particles (Figure 2), accounting for 75.9% of the total PPD-Qs, whereas 6PPD-quinone takes a dominant proportion in runoff water (48.8%) and roadside soil samples (75.7%). Comparatively, the concentrations of 6PPD-quinone in runoff water from Hong Kong varied in the range of 0.21–2.43 μg/L, which is comparable with the level in creeks affected by urban runoff (1.0–3.5 μg/L) but less than the level in creeks affected by the roadway runoff (4.1–6.1 μg/L) in the Los Angeles region.^26^ Tian *et al.* has reported an acute mortality of 6PPD-quinone in coho salmon and with the lethal concentration 50 (LC_50_) of 0.79 μg/L.^26^ Here, we found that 88% of runoff water samples collected from Hong Kong exceeded this value, indicating a potential risk of the runoff water to the aquatic organisms. In addition, the average level of 6PPD-quinone in roadside soils (234 ng/g) was noticed to be higher than that of the dust samples in a parking lot (41.8 ng/g), an interior vehicle (80.9 ng/g), and an indoor house from Guangzhou, China,^27^ implying that roadside soil may represent an important source of exposure of 6PPD-quinone and other PPD-Qs to humans. Besides runoff water, river water could also be contaminated through the transportation of city road runoff into surface waters. A recent monitoring study of the surface water collected from Don River in the Greater Toronto Area, Ontario, during rain events indicated that 6PPD-quinone exhibits a middle flush dynamic, wherein the contaminant loading is sustained with the increasing volumes of cumulative runoff.^28,42^ The concentration of 6PPD-quinone in the surface water ranged from 0.93 to 2.85 μg/L during the 2nd hour to the 44th hour after rain. It should be also noted that IPPD-quinone was widely distributed in the runoff water samples (a detection frequency of 100%). The median level of IPPD-quinone was determined to be 0.56 μg/L, which accounted for 32.6% of the total PPD-Qs in the runoff water. However, there is a lack of available data on its biotoxicity to aquatic organisms. Therefore, studies on its environmental behaviors and ecological effects are anticipated to be conducted in the near future.

**Table 2:** Descriptive Statistics for Detection Frequencies (DF, %) and Concentrations (pg/m^3^ for Air Particles; μg/L for Runoff Water; and ng/g for Roadside Soils) of PPDs and PPD-Qs in Hong Kong

	air particles (*n* = 16)			runoff water (*n* = 9)			roadside soils (*n* = 12)		
compounds	DF	median	range	DF	median	range	DF	median	range
IPPD	100	0.91	0.44–2.73	94	0.01	<IDL–0.21	96	1.13	0.66–24.5
DPPD	81	0.50	<IDL–0.70	56	0.01	0.01–0.02	100	11.8	3.63–84.4
CPPD	75	0.38	<IDL–0.74	100	0.01	<IDL–0.05	92	1.19	0.73–15.4
6PPD	100	1.78	0.82–6.30	100	0.32	0.21–2.71	100	309	31.4–831
DTPD	56	2.86	<IDL–2.88	56	0.01	<IDL–0.01	72	4.82	<IDL–6.78
total PPDs		6.07	1.75–9.41		0.24	0.04–3.00		381	36.3–847
IPPD-Q	94	0.82	<IDL–86.36	100	0.56	0.18–0.95	60	3.06	<IDL–564
DPPD-Q	100	1.91	0.93–95.7	100	0.19	0.11–0.35	100	60.2	2.87–747
CPPD-Q	69	0.17	<IDL–17.5	94	0.06	<IDL–0.31	72	3.12	<IDL–152
6PPD-Q	100	1.18	0.54–13.8	100	1.12	0.21–2.43	100	234	9.50–936
DTPD-Q	63	0.13	<IDL–7.96	50	0.02	0.01–0.82	92	7.94	1.94–107
total PPD-Qs		4.92	2.52–196		2.17	0.74–3.87		395	12.4–1976

Our analyses also provided information on sourcing the proportion of individual PPDs to PPD-Qs. As shown in Table 2, the concentration ratios of DPPD, IPPD, CPPD, and DTPD to the related quinones in roadside soils were 0.20, 0.37, 0.38, and 0.61, respectively. Similar ratios were observed in the detection of runoff water samples. Intriguingly, 6PPD and 6PPD-quinone were found to exhibit distinct patterns among these environmental matrices. The ratio of 6PPD/6PPD-quinone in roadside soils was determined to be 1.32, which was consistent with the results detected in air particles but much higher than that in runoff waters. Our results also revealed the high transformation ratios of DTPD-quinone and IPPD-quinone in air particles. As the formation of PPD-Qs was associated with the presence of environmental oxidants, the high levels of PPD-Qs observed in the air particles may be attributed to a high frequency contact with atmospheric ozone.^26,43^ Collectively, these results indicated the unique distribution and concentration patterns of PPD-Qs, which should be taken into account when estimating their biotoxicity to atmospheric, terrestrial, and aquatic ecosystems.

### 3.3 Human Exposure Assessment

**Figure 3:**
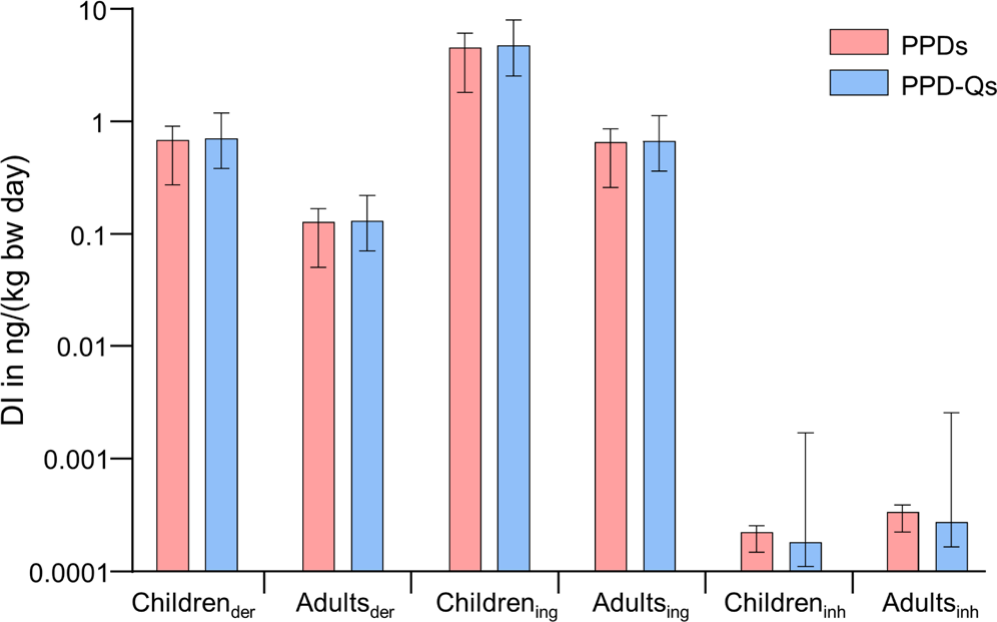
Daily intake rates of PPD antioxidants and PPD-Qs *via* dermal absorption, oral ingestion of roadside soils, and inhalation of outdoor air for adults and children in Hong Kong. The error bars represent the median with a 95% confidence interval of each contaminant.

Since the identified sources of rubber-derived quinones, such as roadside soil, runoff water, and air particles, are closely related to human activities, we anticipate that human exposure to PPD-Qs and their parent compounds *via* diet, inhalation, and dermal absorption is plausible. By the use of the obtained concentrations of PPDs and PPD-Qs in different environmental media, we estimated the daily intake rates of these environmental contaminants for adults and children exposed in Hong Kong. Multiple exposure routes, including the inhalation, ingestion, and dermal absorption, were considered parallelly for the purpose. As shown in Figure 3, the daily intake doses of PPD-Qs are estimated to be 1.08 ng/(kg·day), which exceeded the doses from their parent compounds [0.71 ng/(kg·day)] under the same exposure scenarios. The results indicated that ingestion of roadside soil dust was the main contributor to human exposure of PPDs and PPD-Qs. Dermal absorption represents the second highest exposure pathways, accounting for almost 15% intake rate of oral ingestion. We also noted that the human intakes of PPDs and PPD-Qs *via* inhalation were lower, which may be ascribed to the low levels of these contaminants determined in air particles in Hong Kong. For children, the highest estimated daily doses of these compounds were observed through oral ingestion of roadside soil dust, which were 4.22 ng/(kg·day) for PPDs and 6.35 ng/(kg·day) for PPD-Qs. The total daily intake doses of PPD-Qs for children were estimated to be 7.30 ng/(kg·day), slightly higher than the doses from their parent compounds [4.85 ng/(kg·day)]. Given the extensive use of PPDs and continuous abrasion of rubber-related products, these results imply a potential health risk caused by the transformed quinones of PPD-Qs.

## 4 Environmental Implications

By means of screening analysis using advanced MS techniques, combined with self-synthesized standards for structural elucidation, this study sheds light on the ubiquitous presence of five PPD-Qs in multiple environmental media, including runoff water, roadside soil, and air particles. This phenomenon may be attributed to the extensive production and use of PPDs and related products worldwide. In China, the annual production of PPDs was found to be approximately 100,000 tons in 2010 and that number increased to over 33,000 tons in 2020.^44,45^ The higher production quantities of PPDs, including 6PPD, DPPD, and IPPD, have been reported in the U.S.^15^ Besides, our results reveal distinct fragmentation patterns among these quinones under electrospray conditions, which can be rationalized due to the symmetric/asymmetric substitutions on the moiety of *para*-quinone. The approach can be profitably utilized for the discovery of other rubber-derived quinones. Among the identified quinones, 6PPD-quinone was found to be preserved in the roadway runoff and stormwater-affected creeks and to cause acute mortality of coho salmon (*Oncorhynchus kisutch*).^26^ A recent study by Hiki *et al.* indicated that 6PPD-quinone did not exhibit acute lethal toxicity to freshwater fish (*e.g.*, water flea and Japanese medaka) and crustacean species,^19^ implying the possible species-specific toxicity of this contaminant. However, the biotoxicity of other PPD-Qs to aquatic ecosystems, such as IPPD-quinone and DPPD-quinone, which are abundant in the urban runoff water, remains elusive.

Quantification analyses provide parallel evidence indicating the unique distribution and concentration patterns of the quinones in different environmental compartments. Due to the lack of commercial standards for the newly discovered quinones, particular concerns should be paid to the purities of synthetic standards as they may have a significant impact on the measured environmental concentrations and predicted exposure risks. In addition, given the fact that the quinones exist together in the environment, the effects of both individual and combined PPD-Qs should be considered for investigations on their toxicity and human health risk. We have estimated the daily intakes of total PPD-Qs for adults and children in the compact city of Hong Kong. The results suggest that daily exposure doses of PPD-Qs were higher than the levels of their parent compounds. Considering the prevalence of human indoor activities, particular attention should be paid to evaluate health risks of PPD-Qs for workers in the rubber factory or the places associated with indoor rubber materials such as fitness center flooring.^46^ It should be noted that the identified quinone compounds share the same structural motif with alternated and conjugated C=C and C=O bonds. However, it is still unclear whether these chemicals will be metabolized in living organisms or not. Thus, investigation of possible downstream metabolites of PPD-Qs may provide new insights into their biotoxicity. As a corollary, future research efforts are essentially needed for understanding environmental fates and behaviors and for evaluating ecological and human health risks of the newly discovered quinones.

## Supporting Information Available

Materials and methods, including sample collection and pretreatment procedures and synthesis of PPD-derived quinones; extracted ion chromatograms of the [M + H]^+^ ion of 6PPD-quinone in roadside soil, runoff water, and air particles; screening of PPDs and PPD-derived quinones via a proposed transformation pathway; synthetic route for the preparation of PPD-derived quinones; ^1^H NMR spectra of 6PPD-quinone and 6PPD-quinone-d_5_; peak area ratios between the commercial standard of 6PPD-quinone and the synthesized 6PPD-quinone; SRM chromatograms of five PPD-Qs in roadside soil, runoff water, and air particles; sampling and site information of in roadside soil, runoff water, and air particle samples in Hong Kong; optimized SRM transitions, collision energy values, recoveries, and IQLs for PPDs and PPD-Qs; physiochemical properties of the natural form of PPD antioxidants and their quinones; and parameters used to estimate human exposure to PPDs and PPD-Qs (PDF)
