# Supplementary material for: New Evidence of Rubber-Derived Quinones in Water, Air, and Soil
Source: Environ Sci Technol. 2022 Mar 22;56(7):4142–50. doi: 10.1021/acs.est.1c07376 (PMC8988306; doi:10.1021/acs.est.1c07376)
Supplement: Supplementary file 1 — es1c07376_si_001.pdf [file es1c07376_si_001.pdf]

Supporting information for

New evidence of rubber–derived quinones in water, air, and soil

Guodong Cao[1], Wei Wang[1], Jing Zhang, Pengfei Wu, Xingchen Zhao, Zhu Yang, Di Hu, Zongwei Cai*

State Key Laboratory of Environmental and Biological Analysis, Department of Chemistry, Hong Kong Baptist University, Hong Kong SAR 999077, China

Number of pages: 17

Number of figures: 6

Number of tables: 4

* Corresponding author:

E-mail addresses: zwcai@hkbu.edu.hk (Z. Cai).

[1] The authors contribute equally to this work.

Materials and Methods

Sample collection and pretreatment procedure

Roadside soil samples were collected from green belt areas near the road using a stainless-steel shovel on non-rainy days during August and September 2021 and the sampling location details have been described in Table S1. At least 3 replicate samples from each sampling point were collected and combined into one. The collected samples were transferred to the laboratory within 2 hours. 25-30 grams of samples were weighted, followed by freeze-drying (-40 ℃) and homogeniztion, and then sieved through a 60 mesh for sample extraction.

Air particle samples were collected on the Science Tower in the Campus of Hong Kong Baptist University (~40 m above the ground) during September 4, 2020 to August 20, 2021. A high-volume air sampler (Tisch Environmental Inc., Village of Cleves, OH, USA) was used to collect fine particulate matter ($PM_{2.5}$) onto a quartz fiber filter (20 × 25 cm^2, Whatman, Hillsboro, OR, USA) at a flow rate of 1.13 m^3 min^{-1} for 24 h. The quartz fiber filters were prebaked at 550 °C for 24 h to remove organic contaminants. After sampling, the filters were immediately transferred to the laboratory and stored at -20 ℃ until further analysis. The location details for air particles were provided in Table S1.

Runoff water samples were collected from the small lateral inside drainage canal that drains the roadway surface on the rainy days during August 2021. The details for sampling location, rain events and holding times were provided in Table S1. Around 200 mL samples were collected by using Teflon tubes and stored in coolers on ice during sampling. After completion of the sample collection, 50 mL runoff water sample was immediately filtered by a glass microfiber filter (1.2 um, Whatman, Hillsboro, OR, USA) to remove the possible impurities. The eluted solution was spiked with 50 ng surrogate standard (diphenylamine-d_{10}) and acidified with 2% formic acid by volume, and loaded on a hydrophilic-lipophilic balance solid-phase extraction cartridge (60 mg, 3 mL, Waters). Our result showed that acidification prior to SPE

extraction can slightly improve the recovery for analysis of these targeted chemicals (1%-7%). We speculated that acidification of water may reduce the interference in runoff water samples and thereby attenuate the "matrix effect" in the MS detection. In addition, acidification prior to SPE extraction will lead to protonation of PPDs, which (i.e., ammonium salts) may be more stable than their neutral forms in the water matrix. We also checked the recoveries of quinones from the filters with spiked samples. The results found that the remanent quinones on filters varied in the range of 2.2% to 5.7%, with DTPD-quinone being the highest.

General procedure for the synthesis of PPD-derived quinones

6PPD-quinone and isotope labeled 6PPD-quinone-d_5

1,4-benzoquinone (1.08 g, 10 mmol) was dissolved in hot water (50 mL, 60 °C) followed by adding dropwise to a mixture of aniline (455 μL, 5 mmol) and acetic acid (100 μL, 1.75 mmol) in water (15 mL). The resulting solution was maintained at 60 °C and stirred for 30 min. After completion of the reaction, the crude product was filtered, dried under reduced pressure, and purified by chromatography (*n*-hexane/ dichloromethane = 2:3 to 1:3) with silica gel (200-300 mesh, Qingdao Marine Chemical Factory, China) to afford 2-anilino-1,4-benzoquinone (1.61 g, 8.1 mmol). The compound was characterized as: ^{1}H NMR (400 MHz, DMSO-d_6): δ 9.01 (s, 1H), 7.18-7.42 (m, 5H), 6.82 (d, J = 10.4 Hz, 1H), 6.71 (dd, J = 10.4, 2.4 Hz, 1H), 5.88 (d, J = 2.4 Hz, 1H). ^{13}C NMR (400 MHz, DMSO-d_6): δ 185.9, 183.7, 144.4, 138.8, 138.0, 133.2, 129.3, 125.0, 123.2, 99.6. IR (neat): 3227, 1676, 1631, 1570, 1518, 1497, 1449, 1285, 1080, 867, 861, 731, 696, 615, 508 cm^{-1}; HR-ESI-MS: *m*/*z* calcd for $C_{12}H_{10}NO_2$ [M+H]$^+$ 200.0706, found: 200.0703. To a round-bottomed flask (25 mL) were added 2-anilino-1,4-benzoquinone (200 mg, 1.0 mmol) 1,3-dimethylbutylamine hydrochloride (138 mg, 1.0 mmol), methanol (10 mL) and sodium bicarbonate (100 mg, 1.2 mmol), respectively. The mixture was stirred at room temperature for 4 h. When the reaction is completed, 10 mL of water was added, and the precipitate was filtered and washed by water. The crude product was purified by chromatography (*n*-hexane/ dichloromethane = 1:1 to 2:3) to afford 6PPD-quinone (107 mg,

0.36 mmol). The compound was characterized as: ^{1}H NMR (400 MHz, $CDCl_3$): δ 8.22 (s, 1H), 7.40 (t, *J* = 7.8 Hz, 2H), 7.23 (m, 3H), 6.39 (d, *J* = 8.0 Hz, 1H), 5.97 (s, 1H), 5.43 (s, 1H), 3.55 (m, 1H), 1.67 (m, 1H), 1.53 (m, 1H), 1.38 (m, 1H), 1.22 (d, *J* = 6.4 Hz, 3H), 0.94 (d, *J* = 6.4 Hz, 3H), 0.91 (d, *J* = 6.4 Hz, 3H). ^{13}C NMR (400 MHz, $CDCl_3$): δ 180.1, 178.5, 149.6, 147.7, 137.4, 129.7, 126.1, 122.8, 95.7, 92.8, 46.8, 45.6, 25.2, 22.7, 20.2. IR (neat): 3268, 3232, 2959, 1640, 1558, 1489, 1445, 1355, 1290, 1266, 829, 731, 694, 514 cm^{-1}; HR-ESI-MS: *m/z* = 299.1749 $[C_{18}H_{23}N_2O_2]^+$ (calcd *m/z* = 299.1754). HR-ESI-MS/MS (*m/z*) 256.1197 $[M - C_3H_7]^+$ (calcd *m/z* = 256.1206), 241.0960 $[M - C_4H_{10}]^+$ (calcd *m/z* = 241.0972), 215.0808 $[M - C_6H_{12}]^+$ (calcd *m/z* = 215.0815), 200.0698 $[M - C_6H_{13}N]^+$ (calcd *m/z* = 200.0706), 187.0860 $[M - C_7H_{12}O]^+$ (calcd *m/z* = 187.0866), 100.1118 $[M - C_{12}H_9NO_2]^+$ (calcd *m/z* = 100.1121) and 94.0648 $[M - C_{12}H_{15}NO_2]^+$ (calcd *m/z* = 94.0651). The same procedure was also used for the synthesis of isotope-labeled 6PPD-quinone-d_5 by replacing aniline with aniline-d_5, with a total of (119 mg, 0.39 mmol) 6PPD-quinone-d_5 being obtained, which was characterized as: ^{1}H NMR (400 MHz, $CDCl_3$): δ 8.23 (s, 1H), 6.39 (d, *J* = 8.0 Hz, 1H), 5.97 (s, 1H), 5.43 (s,1H), 3.55 (m, 1H), 1.67 (m, 1H), 1.53 (m, 1H), 1.38 (m, 1H), 1.22 (d, *J* = 6.4 Hz, 3H), 0.94 (d, *J* = 6.4 Hz, 3H), 0.91 (d, *J* = 6.4 Hz, 3H). ^{13}C NMR (400 MHz, $CDCl_3$): δ 180.1, 178.5, 149.6, 147.7, 137.2, 95.7, 92.8, 46.8, 45.6, 25.2, 22.7, 20.2. IR (neat): 3268, 3232, 2959, 1641, 1574, 1552, 1483, 1358, 1288, 1266, 834, 816, 555, 464 cm^{-1}. HR-ESI-MS: *m/z* = 304.2061 $[C_{18}H_{18}D_5N_2O_2]^+$ (calcd *m/z* = 304.2068). HR-ESI-MS/MS (*m/z*) 261.1515 $[M - C_3H_7]^+$ (calcd *m/z* = 256.1519), 246.1280 $[M - C_6H_{12}]^+$ (calcd *m/z* = 246.1285), 220.1125 $[M - C_6H_{12}]^+$ (calcd *m/z* = 220.1129), 205.1017 $[M - C_6H_{11}N]^+$ (calcd *m/z* = 205.1020),192.1176 $[M - C_7H_{12}O]^+$ (calcd *m/z* = 192.1180), 177.0678 $[M - C_7H_{13}NO]^+$ (calcd *m/z* = 177.1071) and 99.0964 $[M - C_{12}H_{10}D_5NO_2]^+$ (calcd *m/z* = 99.0971).

CPPD-quinone and IPPD-quinone

2-anilino-1,4-benzoquinone (200 mg, 1.0 mmol) and methanol (10 mL) were added in a round-

bottomed flask (25 mL). After complete dissolution, a solution of cyclohexylamine was added to it. The solution was prepared by adding cyclohexylamine (115 μL, 1.0 mmol) into methanol (5 mL) and then adding acetic acid (58 μL, 1.0 mmol) under stirring. The mixture was stirred at room temperature under normal atmosphere overnight. After completion of the reaction, the solvent was removed under reduced pressure and the residue was purified by chromatography (*n*-hexane/ dichloromethane = 1:1 to 1:2) to afford CPPD-quinone (119 mg, 0.40 mmol). The compound was characterized as: ^{1}H NMR (400 MHz, $CDCl_3$): δ 8.22 (s, 1H), 7.40 (m, 2H), 7.23 (m, 3H), 6.48 (d, J = 7.2 Hz, 1H), 5.97 (s, 1H), 5.44 (s,1H), 3.30 (m, 1H), 2.04 (m, 2H), 1.80 (m, 2H), 1.66 (m, 1H), 1.32 (m, 5H). ^{13}C NMR (400 MHz, $CDCl_3$): δ 180.3, 178.5, 149.4, 147.7, 137.4, 129.7, 126.0, 122.8, 95.7, 93.1, 51.5, 31.9, 25.5, 24.6. IR (neat): 3262, 3215, 2935, 2921, 1646, 1566, 1508, 1492, 1445, 1358, 1291, 1271, 1210, 818, 737, 693, 492 cm^{-1}. HR-ESI-MS: *m/z* = 297.1569 $[C_{18}H_{21}N_2O_2]^+$ (calcd *m/z* = 297.1598). HR-ESI-MS/MS (*m/z*) 215.0797 $[M - C_6H_{10}]^+$ (calcd *m/z* = 215.0815), 200.0691 $[M - C_6H_{11}N]^+$ (calcd *m/z* = 200.0706), 187.0850 $[M - C_7H_{10}O]^+$ (calcd *m/z* = 187.0866) and 98.0957 $[M - C_{12}H_9NO_2]^+$ (calcd *m/z* = 98.0964). For the synthesis of IPPD-quinone, 2-anilino-1,4-benzoquinone (200 mg, 1.0 mmol), methanol (10 mL) and a solution of isopropyl amine (86 μL, 1 mmol) in methanol with acetic acid (100 μL, 1.75 mmol) were added into a round-bottomed flask (25 mL) and stirred at room temperature overnight. When the reaction is completed, the solvent was removed under reduced pressure and the residue was purified by chromatography (*n*-hexane/ dichloromethane = 2:3 to 1:3) to afford IPPD-quinone (113 mg, 0.44 mmol), which was characterized as: ^{1}H NMR (400 MHz, $CDCl_3$): δ 8.21 (s, 1H), 7.41 (m, 2H), 7.23 (m, 3H), 6.40 (s, 1H), 6.40 (s, 1H), 5.99 (s, 1H), 5.42 (s, 1H), 3.63 (m, 1H), 1.27 (d, J = 6.4 Hz, 6H). ^{13}C NMR (400 MHz, $CDCl_3$): δ 180.2, 178.6, 149.4, 147.6, 137.4, 129.7, 126.1, 122.8, 95.8, 93.3, 44.4, 21.9. IR (neat): 3272, 3235, 1643, 1559, 1488, 1445, 1358, 1291, 1218, 822, 731, 695, 510, 598 cm^{-1}. HR-ESI-MS: *m/z* = 257.1264 $[C_{15}H_{17}N_2O_2]^+$ (calcd *m/z* = 257.1285). HR-ESI-MS/MS (*m/z*) 215.0799 $[M - C_3H_6]^+$ (calcd *m/z* = 215.0815), 200.0693 $[M - C_3H_7N]^+$ (calcd *m/z* = 200.0706),

187.0853 [M - C_4H_6O]$^+$ (calcd *m/z* = 187.0866) and 94.0646 [M - $C_9H_9NO_2$]$^+$ (calcd *m/z* = 94.0651).

DPPD-quinone and DTPD-quinone

Into a round-bottomed flask (50 mL), 1,4-benzoquinone (324 mg, 3 mmol) was dissolved in methanol (20 mL) and then added aniline (182 µL, 2.0 mmol). Formation of a precipitate indicated the reaction progress. The mixture was stirred at room temperature for 1h. When the reaction is completed, the crude product was filtered, washed by methanol, and recrystallized in ethanol to afford DPPD-quinone (192 mg, 0.66 mmol), which was characterized as: IR (neat): 3274, 3227, 3055, 1639, 1566, 1510, 1489, 1443, 1358, 1289, 1232, 1188, 1175, 1094, 1080, 1023, 1003, 895, 859, 842, 762, 741, 725, 695, 622, 510, 498, 482, 469 cm^{-1}. HR-ESI-MS: *m/z* = 291.1120 [$C_{18}H_{15}N_2O_2$]$^+$ (calcd *m/z* = 291.1128). HR-ESI-MS/MS (*m/z*) 263.1172 [M - CO]$^+$ (calcd *m/z* = 263.1179), 235.1224 [M - C_2O_2]$^+$ (calcd *m/z* = 235.1230), 198.0546 [M - C_2O_2]$^+$ (calcd *m/z* = 198.0550), 170.0597 [M - C_7H_7NO]$^+$ (calcd *m/z* = 170.0600), 144.0441 [M - $C_{10}H_7NO_2$]$^+$ (calcd *m/z* = 144.0448), and 94.0649 [M - $C_{12}H_7NO_2$]$^+$ (calcd *m/z* = 94.0651). DTPD-quinone was synthesized by added 1,4-benzoquinone (324 mg, 3 mmol), methanol (20 mL) and o-toluidine (214 µL, 2.0 mmol) in a round-bottomed flask (50 mL), followed by 1 h stirring at room temperature. After completion of the reaction, the solvent was removed under reduced pressure and the crude product was purified by chromatography (*n*-hexane/ dichloromethane = 1:1 to 2:5) to afford DTPD-quinone (146 mg, 0.46 mmol). The compound was characterized as: ^{1}H NMR (400 MHz, $CDCl_3$): δ 7.90 (s, 2H), 7.28 (m, 6H), 7.21 (m, 2H), 5.64 (s, 2H), 2.31 (s, 6H). ^{13}C NMR (400 MHz, $CDCl_3$): δ 180.1, 148.3, 135.4, 133.2, 131.5, 127.2, 124.7, 95.5, 17.9. IR (neat): 3233, 1638, 1605, 1555, 1502, 1455, 1358, 1279, 733, 508, 478 cm^{-1}. HR-ESI-MS: *m/z* = 319.1416 [$C_{20}H_{19}N_2O_2$]$^+$ (calcd *m/z* = 319.1441). HR-ESI-MS/MS (*m/z*) 304.1183 [M – CH_3]$^+$ (calcd *m/z* = 304.1206), 291.1471 [M - CO]$^+$ (calcd *m/z* = 291.1492), 212.0690 [M - C_7H_9N]$^+$ (calcd *m/z* = 212.0706) and 184.0743 [M - C_8H_9NO]$^+$ (calcd

m/z = 184.0757).

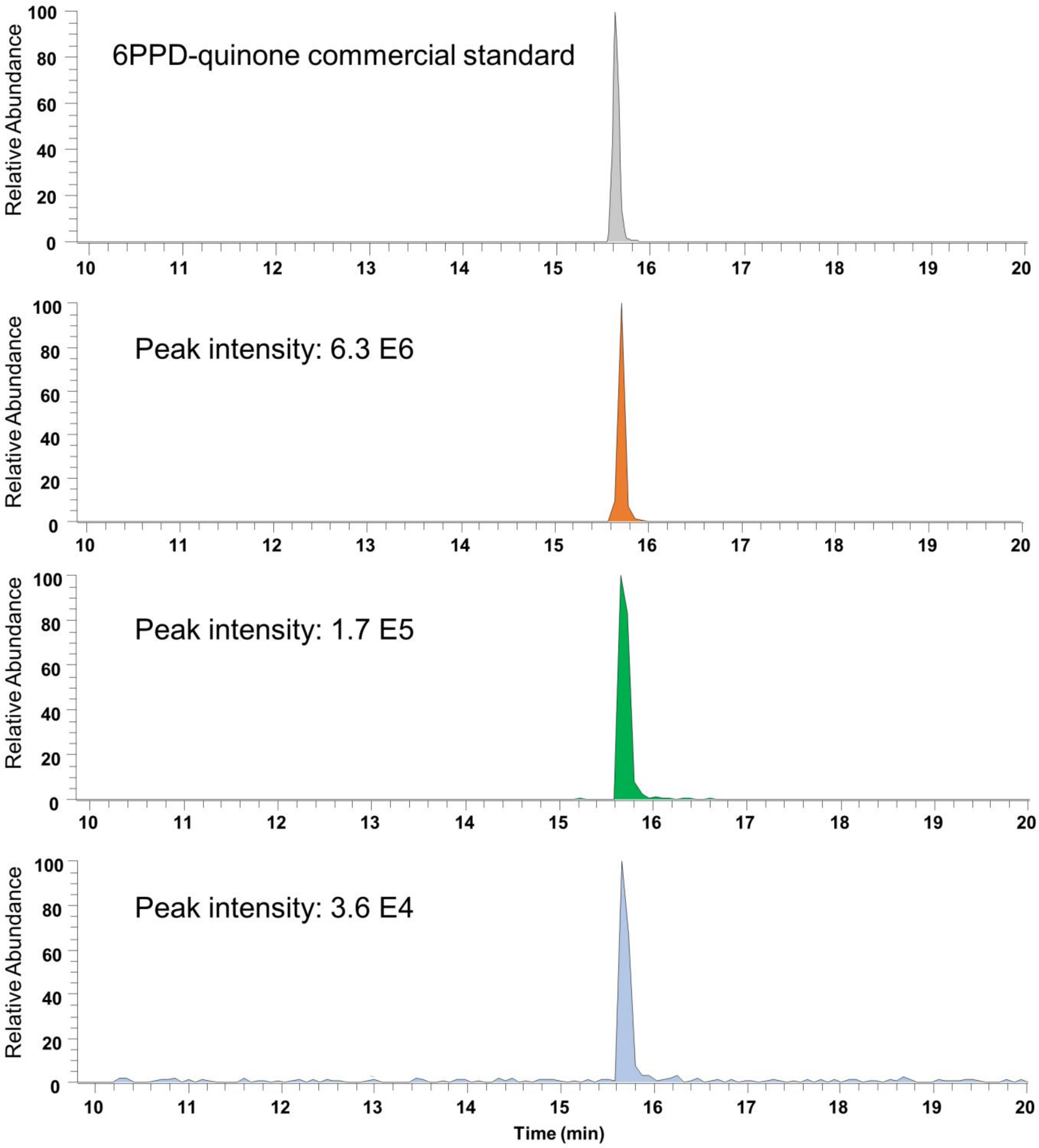

Figure S1. Extracted ion chromatograms of $[M+H]^+$ ion of 6PPD-quinone in roadside soil (orange), runoff water (green) and air particle (cyan) in comparison to the commercial standard of 6PPD-quinone (gray).

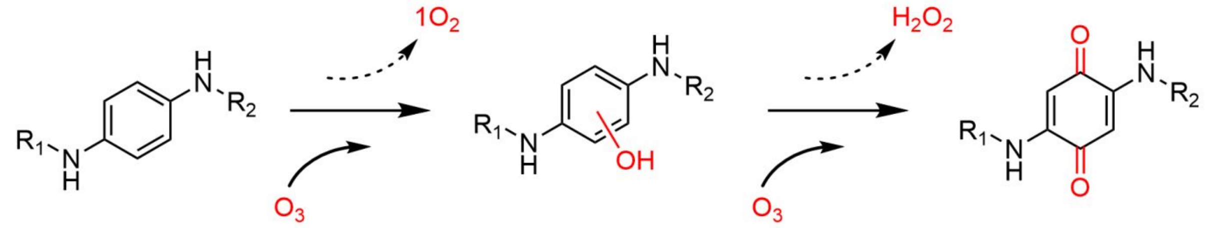

R_1	R_2		R_1	R_2	
Phenyl	Isopropyl	IPPD	Phenyl	Cyclohexyl	CPPD
Phenyl	Phenyl	DPPD	Phenyl	1,3-dimethylbutyl	6PPD
Phenyl	1,4-dimethylamyl	7PPD	2-methylphenyl	2-methylphenyl	DTPD
Phenyl	1-methylheptyl	8PPD	2-naphtyl	2-naphtyl	DNPD

Figure S2. Screening of PPDs and PPDs-derived quinones via a proposed transformation pathway[1].

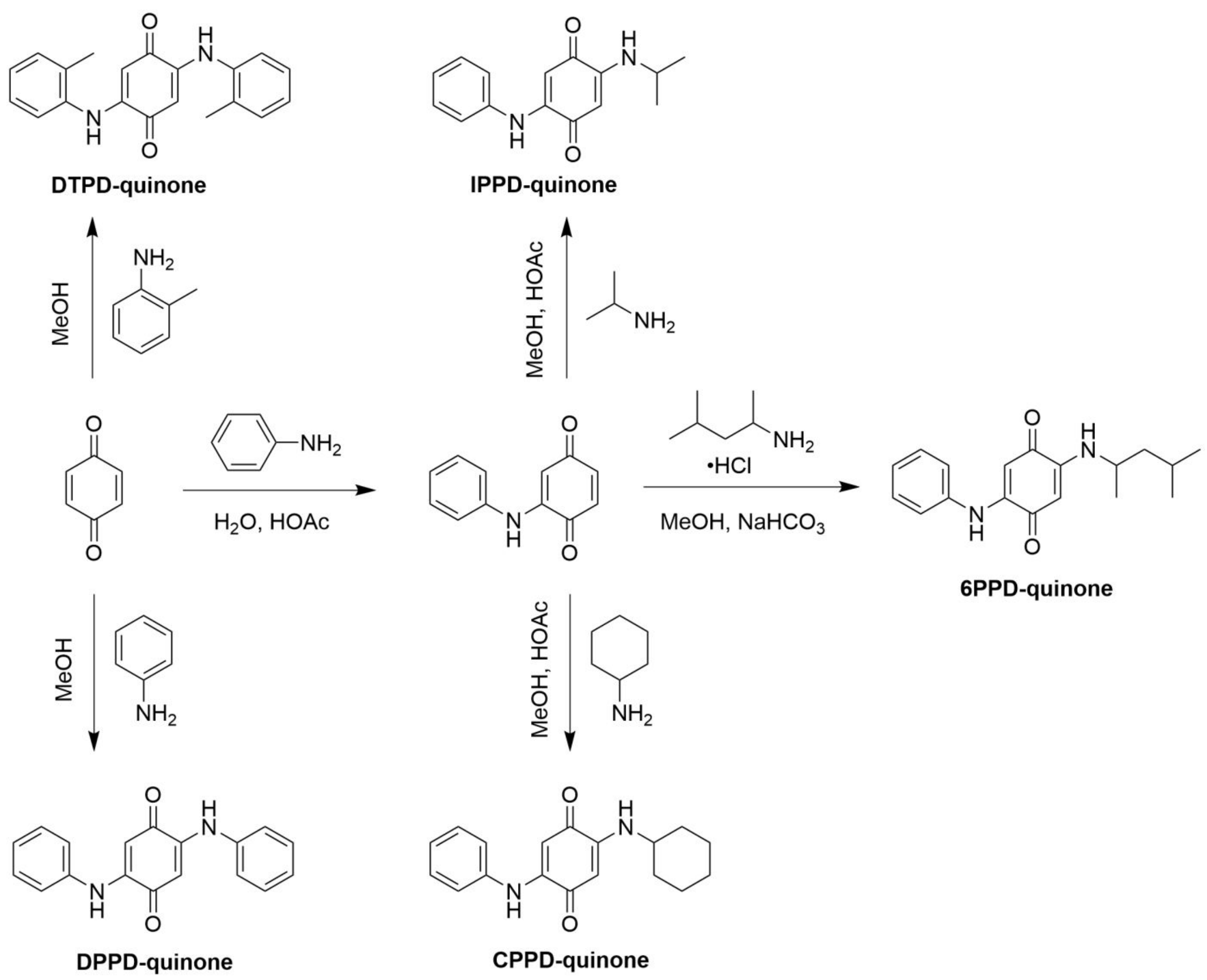

Figure S3. Synthetic route for preparation of PPDs-derived quinones.

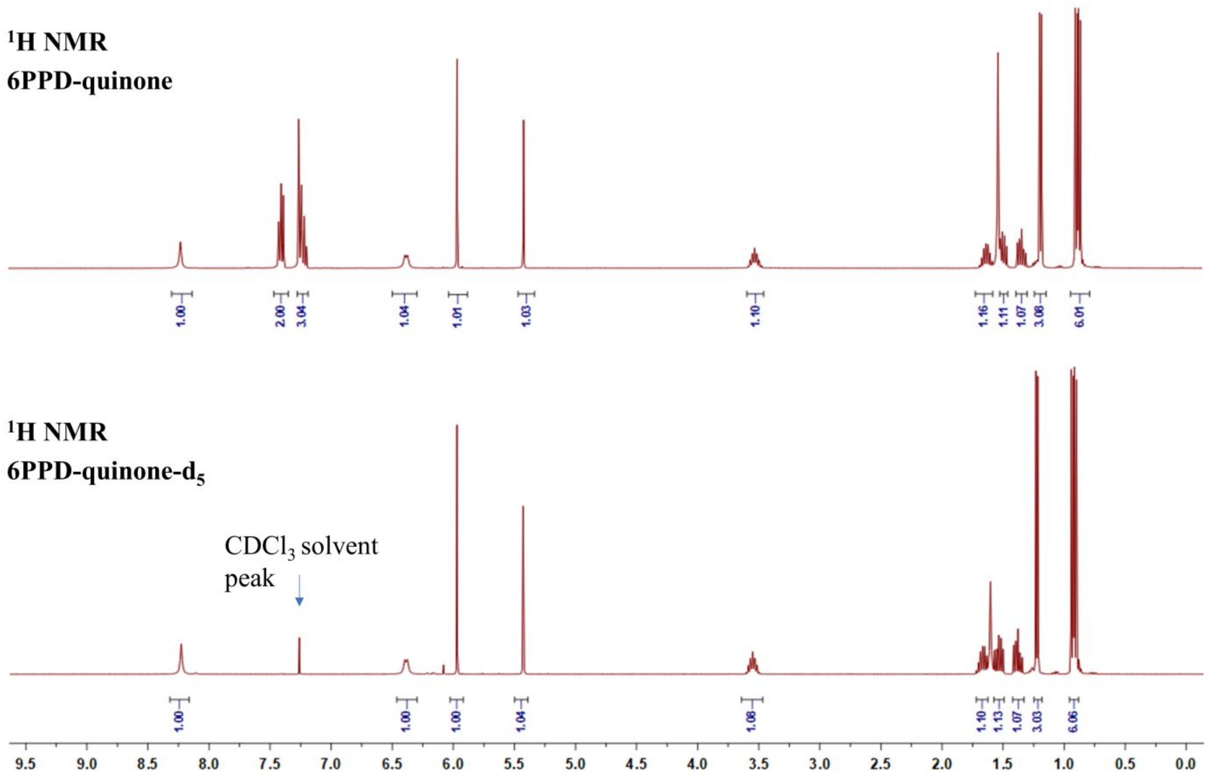

Figure S4. ^{1}H NMR spectra of 6PPD-quinone (upper) and 6PPD-quinone-d_5 (lower).

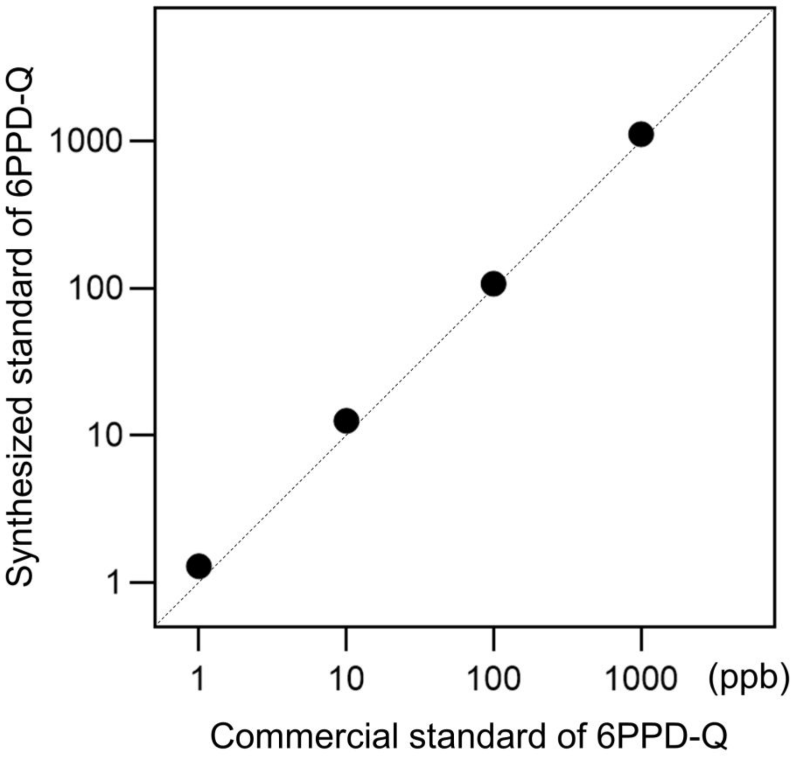

Figure S5. Peak area ratios between commercial standard of 6PPD-quinone and the synthesized 6PPD-quinone. The serially diluted solutions were prepared at 1, 10, 100 and 1000 ppb and determined by UHPLC-TSQ-MS.

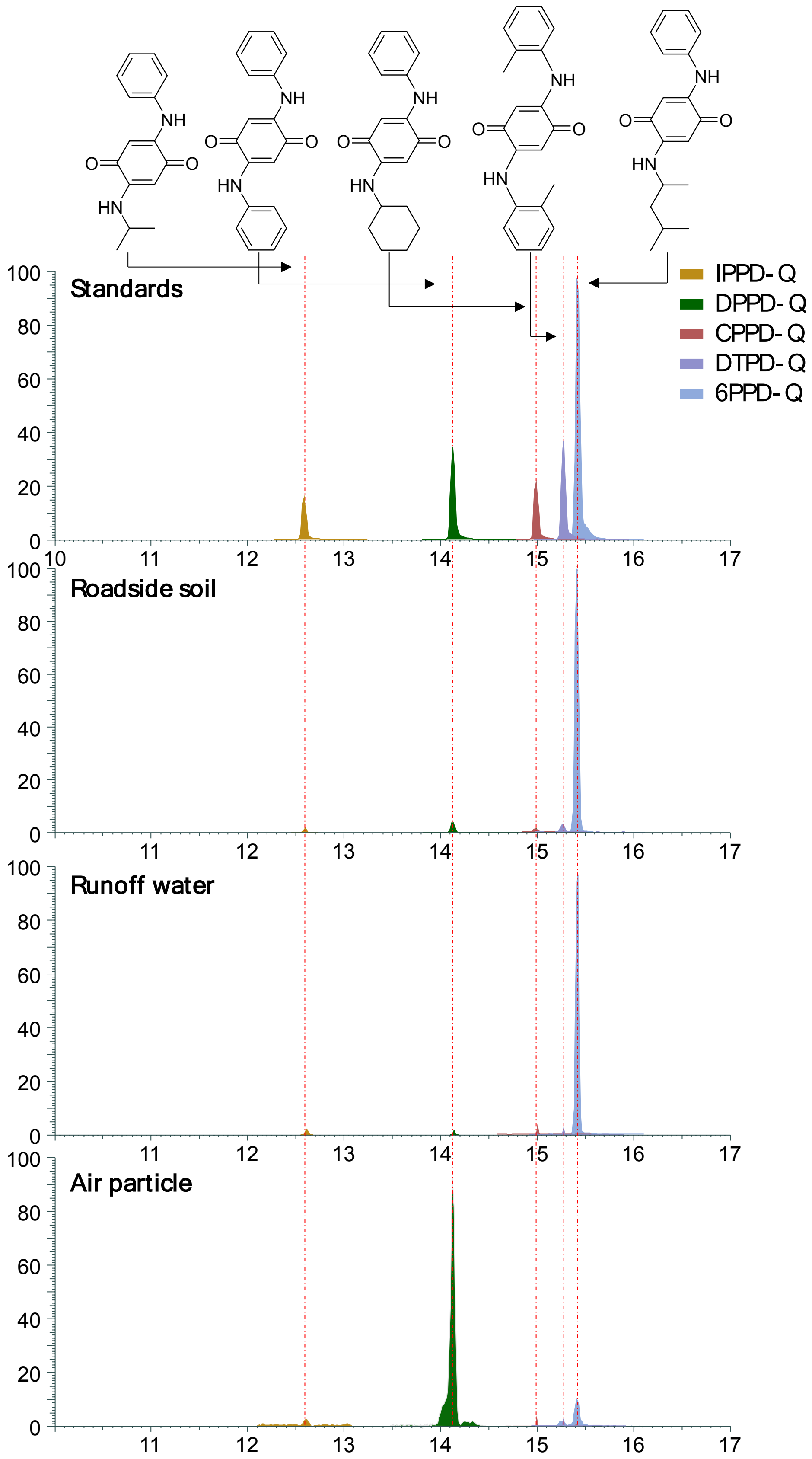

Figure S6. SRM chromatograms of five PPD-Qs in roadside soil, runoff water and air particle in comparison to the synthesized standards.

Table S1. Sampling and site information of runoff water (RW), roadside soil (RS), and air particle (AP) samples in Hong Kong. The roadside soil and runoff water samples were sampled at the same site twice at different time.

Sample type	Site No.	Sampling date	Latitude	Longitude
Air particle	BU-1	9/4/2020	22.338150	114.185116
Air particle	BU-2	9/28/2020	22.338150	114.185116
Air particle	BU-3	11/9/2020	22.338150	114.185116
Air particle	BU-4	11/27/2020	22.338150	114.185116
Air particle	BU-5	12/3/2020	22.338150	114.185116
Air particle	BU-6	12/29/2020	22.338150	114.185116
Air particle	BU-7	1/8/2021	22.338150	114.185116
Air particle	BU-8	1/26/2021	22.338150	114.185116
Air particle	BU-9	2/1/2021	22.338150	114.185116
Air particle	BU-10	2/25/2021	22.338150	114.185116
Air particle	BU-11	3/3/2021	22.338150	114.185116
Air particle	BU-12	3/27/2021	22.338150	114.185116
Air particle	BU-13	4/8/2021	22.338150	114.185116
Air particle	BU-14	4/26/2021	22.338150	114.185116
Air particle	BU-15	5/14/2021	22.338150	114.185116
Air particle	BU-16	8/20/2021	22.338150	114.185116
Runoff water	RW-1	8/19/2021[a], 8/24/2021[b]	22.336963	114.185700
Runoff water	RW-2	8/19/2021, 8/24/2021	22.336960	114.186149
Runoff water	RW-3	8/19/2021, 8/24/2021	22.336403	114.186688
Runoff water	RW-4	8/19/2021, 8/24/2021	22.337163	114.187714
Runoff water	RW-5	8/19/2021, 8/24/2021	22.336852	114.187982
Runoff water	RW-6	8/19/2021, 8/24/2021	22.336383	114.185613
Runoff water	RW-7	8/19/2021, 8/24/2021	22.336433	114.184411
Runoff water	RW-8	8/19/2021, 8/24/2021	22.337341	114.184082
Runoff water	RW-9	8/19/2021, 8/24/2021	22.337311	114.182007
Roadside soil	RS-1	8/18/2021, 9/7/2021	22.381619	114.197933
Roadside soil	RS-2	8/18/2021, 9/7/2021	22.381780	114.198270
Roadside soil	RS-3	8/18/2021, 9/7/2021	22.381944	114.198209
Roadside soil	RS-4	8/18/2021, 9/7/2021	22.381239	114.197506
Roadside soil	RS-5	8/18/2021, 9/7/2021	22.379864	114.196653
Roadside soil	RS-6	8/18/2021, 9/7/2021	22.376773	114.197559
Roadside soil	RS-7	8/18/2021, 9/7/2021	22.376773	114.197559
Roadside soil	RS-8	8/18/2021, 9/7/2021	22.379277	114.196341
Roadside soil	RS-9	8/18/2021, 9/7/2021	22.375579	114.191556
Roadside soil	RS-10	8/18/2021, 9/7/2021	22.374762	114.186507
Roadside soil	RS-11	8/18/2021, 9/7/2021	22.337363	114.184783
Roadside soil	RS-12	8/18/2021, 9/7/2021	22.337315	114.182731

Note: The holding times of rain event **a** and **b** were approximately 60 and 45 min, respectively.

Table S2. Optimized SRM transitions, collision energy (CE) values, recoveries instrument quantification limits (IQLs) for PPDs and PPD-Qs.

Compound	RT (min)	Precursor ion (*m/z*)	CE (eV)	Equations	Qualifier ions (*m/z*)	Recovery (%)				IQLs (ng/mL)
						Water[a]	Water[b]	Soil	Air particle	
IPPD	9.02	227.2	26	Y =-0.00227+0.228*X	212.1; 184.1	78±5	77±6	101±14	79±6	0.031
DPPD	15.99	261.1	26	Y =-0.00746+0.159*X	184.1; 169.1	85±3	89±11	89±3	96±14	0.055
CPPD	10.55	267.2	22	Y =-0.000495+0.0578*X	223.1; 185.1	84±1	73±5	77±9	91±3	0.044
6PPD	11.10	269.2	32	Y =-0.000365+0.0226*X	212.1; 93.1	86±1	77±11	94±3	82±3	0.035
DTPD	17.51	289.2	23	Y =-0.0168+0.331*X	198.1; 183.1	82±5	75±9	70±3	81±11	0.296
IPPD-Q	12.77	257.1	24	Y = 0.0000115+0.00852*X	215.1; 187.1	93±1	84±4	74±7	84±4	0.019
DPPD-Q	14.30	291.1	20	Y = 0.004425+0.0303*X	235.1; 263.1	75±3	77±6	113±4	78±4	0.055
CPPD-Q	15.24	297.2	28	Y =-0.0000167+0.00931*X	215.1; 187.1	92±1	84±5	109±7	74±3	0.041
6PPD-Q	15.68	299.2	26	Y = 0.00167+0.0473*X	241.1; 215.1	86±1	77±7	83±8	91±13	0.023
DTPD-Q	15.46	319.1	27	Y = 0.0000529+0.0179*X	212.1; 184.1	83±1	81±9	104±7	86±1	0.020

a and **b** represent water recoveries with and without matrix, respectively.

Table S3. Physicochemical properties of the natural form of PPDs antioxidants and their quinones were calculated by EPI Suite software[2], and their total persistent and transport potential were estimated by Marvin (V15.6.29 Chemxon, US), and Pov-LRTR[3]. ($LogK_{OA}$: octanol-air partitioning coefficient; $LogK_{OC}$: soil organic carbon-water partitioning Coefficient; POV: overall persistence).

**Compound**	**Water solubility (mg/L, 25 ℃)**	**$LogK_{OA}$**	**Vapor Pressure (Pa, 25 ℃)**	**$LogK_{OC}$**	**pKa_1**	**pKa_2**	**POV (day)**
IPPD	50.32	10.510	2.76E-02	3.636	6.42	0.58	105
DPPD	1.277	13.007	1.27E-05	4.715	2.42	-0.11	108
CPPD	2.083	11.858	2.07E-03	3.854	6.42	0.58	108
6PPD	2.8262	11.332	5.99E-03	4.363	6.46	0.58	108
DTPD	0.5914	13.122	3.33E-04	5.143	2.38	-0.15	108
IPPD-Q	1400	14.288	9.71E-04	3.202	0.87	-0.78	96
CPPD-Q	56.93	15.635	6.80E-05	3.419	0.62	0.59	107
DPPD-Q	15.38	16.014	3.99E-05	3.758	0.47	-0.84	106
6PPD-Q	51.34	15.319	2.03E-04	3.928	0.61	0.59	107
DTPD-Q	1.225	17.028	1.05E-05	4.187	0.52	-0.79	172

Table S4. Parameters used to estimate human exposure to PPDs and PPD-Qs[4-5].

**Parameters**	**Unit**	**Children**	**Adults**
Ingestion rate (IR_{ing})	mg/day	200	100
Inhalation rate (IR_{inh})	m^3/day	5.65	16.7
Body weight (BW)	kg	16.58	58.55
Exposure frequency (EF)	days/year	365	365
Exposure duration (ED)	years	6	24
Average time during exposure (AT)	day	365×70	365×70
Conversion factor (CF)	kg/mg	10^{-6}	10^{-6}
Exposure time (ET)	h/day	0.08	0.195
Skin surface area available for contact (SA)	cm^2	1150	2145
Soil to skin adherence factor (AF)	mg/cm^2	0.2	0.07
Adsorption factor (ABS)	%	13	13

References

1. Tian, Z.; Zhao, H.; Peter, K. T.; Gonzalez, M.; Wetzel, J.; Wu, C.; Hu, X.; Prat, J.; Mudrock, E.; Hettinger, R., A ubiquitous tire rubber–derived chemical induces acute mortality in coho salmon. *Science* **2021,** *371* (6525), 185-189.
2. U.S. EPA. EPI Suite™-Estimation Program Interface (Version 4.11). https://www.epa.gov/tsca-screening-tools/epi-suitetm-estimation-program-interface (accessed August 23, 2021).
3. OECD Pov and LRTP Screening Tool. https://www.oecd.org/chemicalsafety/risk-assessment/oecdpovandlrtpscreeningtool.htm (accessed August 23, 2021).
4. Zhang, J.; Zhang, X.; Wu, L.; Wang, T.; Zhao, J.; Zhang, Y.; Men, Z.; Mao, H., Occurrence of benzothiazole and its derivates in tire wear, road dust, and roadside soil. *Chemosphere* **2018,** *201*, 310-317.
5. Ji, Y.; Wang, F.; Zhang, L.; Shan, C.; Bai, Z.; Sun, Z.; Liu, L.; Shen, B., A comprehensive assessment of human exposure to phthalates from environmental media and food in Tianjin, China. *J Hazard Mater* **2014,** *279*, 133-40.
